# Frailty Predicts Neurological Outcome in Chronic Subdural Hematoma: A Single-Center Prospective Cohort Study

**DOI:** 10.3390/geriatrics11030062

**Published:** 2026-05-19

**Authors:** Tobias Philip Schmidt, Christian Jacquemain, Jule Rupprecht, Kerstin Jütten, Laura Schlager, Christian Blume, Michael Veldeman, Hans Clusmann, Anke Höllig, Catharina Conzen-Dilger

**Affiliations:** Department of Neurosurgery, Rheinisch-Westfälische Technische Hochschule (RWTH) Aachen University, 52074 Aachen, North Rhine-Westphalia, Germany

**Keywords:** frailty, chronic subdural hematoma, neurological outcome, clinical frailty scale, geriatric

## Abstract

Objectives: Frailty, reflecting reduced physiological reserve, has emerged as a predictor of postoperative outcomes in neurosurgery and may provide greater prognostic value than age. In chronic subdural hematoma (cSDH), prospective data remain scarce. This study investigates the association between preoperative frailty, assessed using the Clinical Frailty Scale (CFS), and postoperative recovery in cSDH patients. Methods: In this ongoing prospective single-center cohort study, 78 consecutive patients (≥60 years) with cSDH were enrolled between August 2022 and October 2024. After exclusion of four conservatively managed patients, 74 surgically treated patients were included in the analysis. Frailty was defined as Clinical Frailty Scale (CFS) ≥ 5. The primary outcome was the Glasgow Outcome Scale-Extended at 6 months (GOSE6). Secondary outcomes included GOSE at discharge (GOSE0) and three months (GOSE3), revision surgery, intensive care unit (ICU) admission, and mortality after six months. Results: Higher CFS scores significantly correlated with poorer outcome at 6 months (r = −0.68, *p*_adj_ = 0.011). In regression analysis, frailty (*p* < 0.001), age (*p*_adj_ = 0.014), and revision surgery (p_adj_ = 0.009) were significant predictors of outcome. Frailty was associated with a reduced likelihood of a good neurological outcome (OR = 0.02, 95% CI: [0.004, 0.085]). Frail patients had significantly poorer outcomes at all timepoints (all *p*_adj_ = 0.014) and none achieved a favorable outcome (GOSE ≥ 6). Six-month mortality was significantly higher in frail patients compared to non-frail patients (32% vs. 4%, *p*_adj_ = 0.048, relative risk RR = 3.29, 95% CI [1.67, 5.78]). Conclusions: Our interim results suggest that preoperative frailty, as measured by the CFS, is strongly associated with poorer neurological recovery and higher mortality following surgical treatment of cSDH. Frailty assessment may facilitate individualized treatment strategies and improve risk stratification beyond age or comorbidity burden.

## 1. Introduction

Chronic subdural hematoma (cSDH) is a common neurosurgical condition predominantly affecting the elderly population, with its incidence steadily rising due to the aging population [1,2]. This demographic shift highlights the growing need for an improved understanding of risk factors that contribute to worse outcomes after surgical treatment of cSDH in this population [1]. Although advanced age itself is an established risk factor, it insufficiently captures the complex interplay of age-related physiological decline, comorbidities, and functional impairments that define frailty.

Frailty is a multidimensional construct encompassing diminished physiological reserve and increased vulnerability to stressors, which is often exacerbated by comorbidities such as cardiovascular disease, diabetes, and polypharmacy. These conditions are prevalent in cSDH patients and can complicate both surgical and postoperative management. Unlike chronological age, frailty provides a more nuanced assessment of a patient’s overall health status, making it a valuable metric for risk stratification [3,4].

Frailty can be assessed using various tools, including the Clinical Frailty Scale (CFS), Fried’s Frailty Phenotype, and the Frailty Index. While Fried’s criteria and the Frailty Index offer detailed multidimensional evaluations, they are labor-intensive and time-consuming [5]. The CFS, by contrast, provides a rapid, clinically feasible assessment with promising predictive value in elderly neurosurgical populations and can also be reliably applied through collateral history when direct patient evaluation is limited [6].

The pathophysiological processes underlying cSDH, including impaired hemostasis, inflammation, and neoangiogenesis, are influenced by the patient’s systemic resilience [7,8]. Frailty-associated deficits in wound healing and repair mechanisms may exacerbate these processes, contributing to an increased likelihood of hematoma recurrence and delayed recovery [8].

Existing classification systems for cSDH, such as Nakaguchi’s hematoma type classification or the internal architecture subtypes by Hamou et al., aim to predict recurrence risk based on anatomical and radiological findings [9,10]. However, these models do not account for patient-specific factors such as systemic resilience and physiological reserve. Incorporating frailty assessments into current risk prediction frameworks could enhance the precision of outcome predictions and facilitate more personalized treatment strategies [8]. By acknowledging the broader context of a patient’s health beyond their radiological presentation, frailty evaluations may bridge the gap between anatomical classifications and holistic patient care.

Various retrospective studies have underscored the significant impact of frailty on outcomes in patients with cSDH [11,12]. Recently, a systematic review by Pahwa et al. (2024) found that frailty was consistently associated with adverse outcomes, including increased mortality, complications, and recurrence [8]. Collectively, these findings highlight the necessity for prospective studies to comprehensively assess the relationship between frailty and outcomes in cSDH patients.

Our ongoing prospective cohort study aims to address this gap by employing the validated CFS to quantify the influence of frailty on clinical outcomes following cSDH treatment. By doing so, we seek to provide evidence that supports the integration of frailty assessments into clinical decision-making processes, ultimately contributing to more personalized treatment strategies and improved patient care.

## 2. Materials and Methods

### 2.1. Data Aquisition

**Figure 1:**
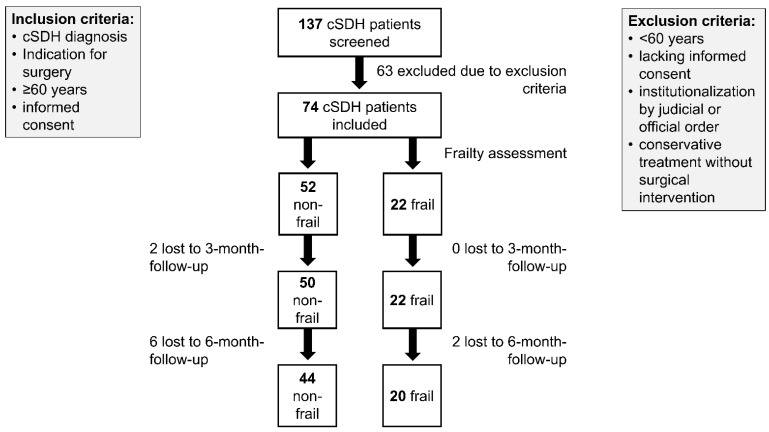
Flowchart demonstrating inclusion and exclusion parameters of patients eligible for study participation. Abbreviation: cSDH = chronic subdural hematoma.

This ongoing prospective study received approval from the local ethics committee of the Medical Faculty at RWTH Aachen University (EK 382/21, 6 July 2022). All included participants provided written informed consent, in line with the Declaration of Helsinki (Medical Association 2008, last revision 2024). Exclusion criteria included patients younger than 60 years, lacking informed consent, or institutionalization by judicial or official order, or conservative treatment without surgical intervention (see Figure 1). This analysis provides an interim report based on 78 patients enrolled between August 2022 and October 2024. All study participants underwent neurological examination and frailty assessment according to the CFS. The pictogram-based scale enables the classification of frailty into nine distinct levels, from “very fit” to “terminally ill”. The scale demonstrated high assessment reliability and minimal interrater bias [6]. Each patient was given a score based on their everyday performance before initial subdural bleeding. Patient treatment was carried out independently of study participation in accordance with clinical guidelines and the established standard algorithms of the Neurosurgical Department at RWTH Aachen University Hospital.

### 2.2. Treatment Algorithm

As previously described, cSDHs were surgically treated upon diagnosis if they caused neurological deficits such as paresis, gait disturbances, speech impairments, or seizures [10,13]. Isolated headaches were not considered an indication for surgery. Asymptomatic cSDHs were managed conservatively unless imaging showed significant mass effect, including sulcal effacement, ventricle compression, or midline shift. The primary surgical approach was burr hole craniotomy with one or two non-suction subdural drains placed after hematoma evacuation. The choice of procedure was based on surgeon’s preference. Subdural drains remained in place for 1 to 3 days. Patients were discharged once symptoms resolved or if postoperative imaging showed no significant residual mass. Follow-up imaging was routinely conducted approximately 21 days after surgery. Asymptomatic hematomas without mass effects were monitored in three- to four-week intervals until resolution or until symptoms or mass effect warranted surgery. Recurrence was defined as an increase in hematoma volume with signs of mass effect or the reappearance of neurological deficits, requiring reintervention. Follow-up CT scans continued until full remission was achieved. Hematoma size was measured as the largest width on the last axial CT imaging indicating surgical treatment. Follow-up assessments were conducted at three- and six-month post-discharge, either through clinical visits or structured telephone interviews.

### 2.3. Statistical Analyses

The primary outcome was the Glasgow Outcome Scale—Extended (GOSE) six months post-discharge (GOSE6) [14]. Secondary outcomes included GOSE at discharge (GOSE0) and at three months (GOSE3), as well as length of hospital stay, discharge destination, revision surgery, need for ICU, and mortality.

All statistical analyses were performed with R [ R Core Team (2022). R: A language and environment for statistical computing. R Foundation for Statistical Computing, Vienna, Austria. URL https://www.R-project.org/ (accessed on 7 April 2026).], RStudio 2025.09.1 [Posit team (2025). RStudio: Integrated Development Environment for R. Posit Software, PBC, Boston, MA. URL http://www.posit.co/ (accessed on 7 April 2026).], and Jamovi 2.3 [The jamovi project (2022). jamovi. (Version 2.3) [Computer Software]. Retrieved from URL https://www.jamovi.org (accessed on 7 April 2026).].

Participants’ demographics and clinical characteristics were explored using descriptive statistics. Interval and ordinal data (age and length of stay, Charlson Comorbidity Index [CCI], GOSE0, GOSE3, and GOSE6) were summarized with median and interquartile range (–IQR) and nominal variables (gender, hypertension, diabetes mellitus, smoking, antiplatelet medication and anticoagulant use, need for intensive care unit (ICU), revision surgery, discharge place, and in-house mortality) were reported as the count and percentage. The association between patients’ age, CFS, CCI and neurological outcome (GOSE0, GOSE3, and GOSE6) was explored by computing Spearman’s rank correlation coefficient, testing two-sided with a significance level set to *p* < 0.05, and applying Bonferroni–Holm correction for multiple comparisons. Only adjusted *p*-values (p_adj_) will be reported. Frailty-related group differences were explored using the Mann–Whitney U test (in case of age, length of stay, GOSE0, GOSE3, and GOSE6) and Fisher’s exact test (in case of gender, hypertension, diabetes mellitus, smoking, antiplatelet medication and anticoagulant use, ICU, and revision surgery), testing two-sided with a significance level set to *p* < 0.05, and applying Bonferroni–Holm correction for multiple comparisons. Only adjusted *p*-values will be described, and effect sizes (Mann–Whitney U Test: rank biserial correlation *r_rb_*, Fisher’s exact test: Odds ratios) and their 95% confidence intervals (CIs) will be provided. A binomial logistic regression was performed to determine the effect of frailty, age, antiplatelet, anticoagulation, and revision surgery and predict the likelihood of a good neurological outcome six months post-discharge, testing two-sided with a significance level set to *p* < 0.05. All figures and graphical representations were generated using GraphPad Prism (Version 11.0.0 for Windows, GraphPad Software, Boston, MA, USA, www.graphpad.com) and R.

### 2.4. Missing Data

Our final data set revealed missing data regarding the primary outcome variable neurological outcome at six months (14% [n = 10] of the included patients [N = 74] were lost of follow-up). This issue was addressed by statistically testing the mechanism underlying missing data, to ensure data were not missing systematically across variables of interests. To do so, missing data were coded in a separate variable, a missing data indicator (data complete: 0 = no, 1 = yes), and compared across frailty groups by means of Fisher’s exact test, revealing no significant association between frailty and missingness (p_adj_ = 0.394, OR = 1.82, 95% CI [0.35, 9.35]). In addition, the missing data indicator was entered in a binominal logistic regression to predict the likelihood of missing data and the effect of being frail (among age, antiplatelet, anticoagulation, and revision surgery). The regression model was not significant (χ^2^ = 519; *p*_adj_ = 0.394), and results further showed no relations at all between the tested variables, allowing the assumption that missing data were completely missing at random. For this reason, complete case analysis was applied to address missing data [15].

## 3. Results

### 3.1. Study Population

A total of 78 consecutive patients with cSDH were prospectively enrolled between August 2022 and October 2024. Four patients were excluded due to conservative (non-surgical) management, leaving N = 74 included patients for subsequent analyses. The cohort was further stratified by CFS scores into a “frail” group (defined as CFS score ≥ 5, N_frail_ = 22) and a “non-frail” group (CFS score < 5, N_non-frail_ = 52). At six months post-discharge, 14% (n = 10) of the included patients were lost to follow-up, leaving n = 64 patients included for follow-up analyses.

### 3.2. Demographics

The cohort was predominantly male (69%, n = 51, N = 74), with a median age of 76.5 years (IQR 67.3–82.0). Twenty-nine percent (n = 22, N = 74) of the patients revealed a CFS score ≥ 5, and 39% (n = 29, N = 74) a CCI ≥ 3. Overall, 74% (n = 55, N = 74) had a history of arterial hypertension, 22% (n = 16, N = 74) patients suffered from diabetes, and 16% (n = 12, N = 74) were smokers. In addition, 41% (n = 30, N = 74) had antiplatelet medication and 15% (n = 11, N = 74) anticoagulating agents in their medical history. See Table 1 for detailed demographic information.

Median length of stay was five days (IQR 4–8), and overall revision rate was 24% (n = 18, N = 74), with 18 patients transferred to ICU (24%, N = 74). Sixty-eight percent (n = 50, N = 74) of the patients were discharged home. In-hospital mortality was 3% (n = 2, N = 74). See Table 2 for detailed information concerning clinical course, postoperative complications, and discharge disposition.

### 3.3. Association Between Frailty, Charlson Comorbidity Index, Age and Neurological Outcome

Spearman’s rank correlation analysis revealed a significant negative correlation between frailty and neurological outcome (CSF-GOSE0: *r* = −0.501, *p*_adj_ = 0.011; CFS-GOSE3: *r* = −0.674, *p*_adj_ = 0.011; and CFS-GOSE6: *r* = −0.677, *p*_adj_ = 0.012), indicating that higher frailty scores were associated with poorer outcome. Similarly, the CCI correlated significantly with neurological outcome (CCI-GOSE0: *r* = −0.416, *p*_adj_ = 0.011; CCI-GOSE3: *r* = −0.416, *p*_adj_ = 0.011; and CCI-GOSE6: *r* = −0.437, *p*_adj_ = 0.012), as did age (age-GOSE0: *r* = −0.320, *p*_adj_ = 0.015; age-GOSE3: *r* = −0.383, *p*_adj_ = 0.011; and age-GOSE6: *r* = −0.344, *p*_adj_ = 0.015), suggesting that patients with a higher CCI or older age revealed a poorer outcome. Overall, the association between neurological outcome and CFS was strongest, as revealed in higher effect sizes, followed by CCI and age. While CCI revealed a high positive correlation with age (*r* = 0.623, *p*_adj_ = 0.011), CFS and age correlated weakly, yet still remained significant (*r* = 0.235, *p*_adj_ = 0.044).

**Figure 2:**
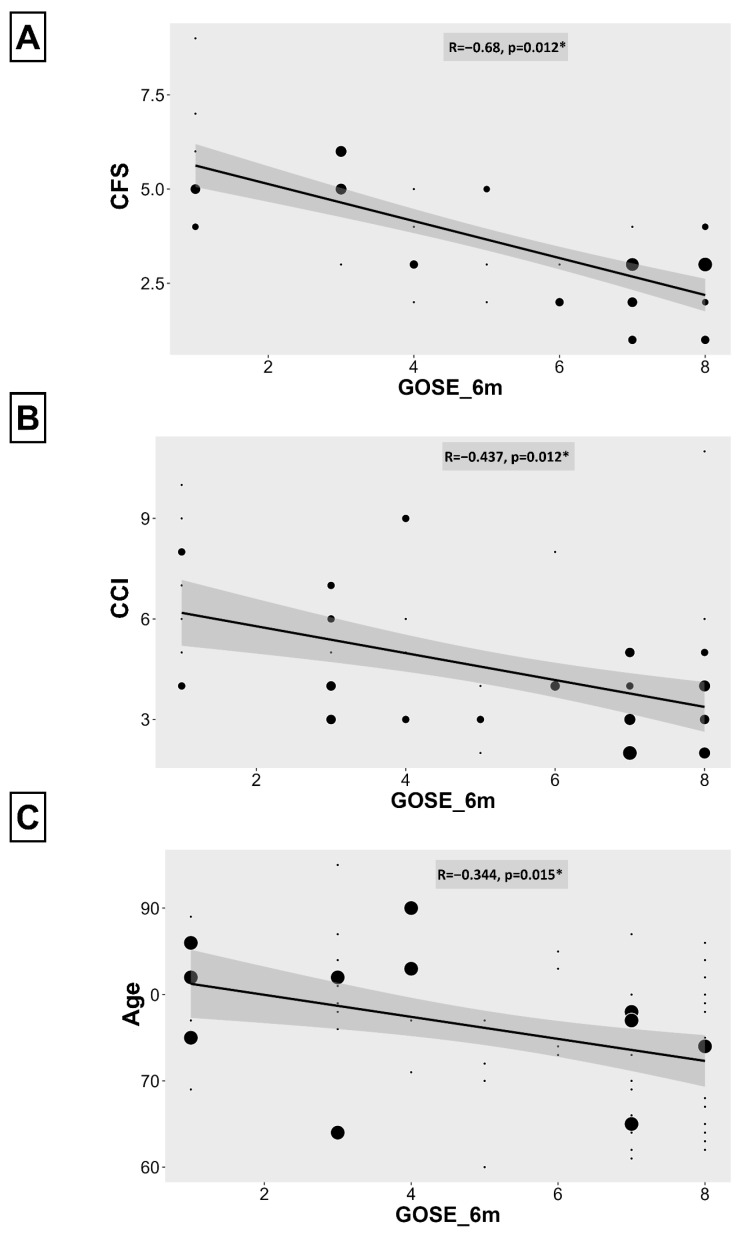
Scatterplots with regression lines showing the association of (**A**) CFS, (**B**) age, and (**C**) CCI with the GOSE at 6 months. Dot size varies according to the number of patients, with bigger dots indicating more and smaller dots indicating less patients. All three parameters demonstrated significant negative correlations with GOSE (CFS: *p*_adj_ = 0.011; age: *p*_adj_ = 0.011; CCI: *p*_adj_ = 0.011). CFS = Clinical Frailty Scale. CCI = Charlson Comorbidity Index. GOSE = Glasgow Outcome Scale-extended. *: *p* < 0.05.

See Figure 2 for six-month correlation plots for CFS, CCI and age.

### 3.4. Subgroup Analysis Frail Versus Non-Frail

No significant differences in age were found between frail (median = 78.5 years, IQR 72.8–82.8) and non-frail patients (median = 74.0 years, IQR 66.0–82.0), as revealed by Mann–Whitney U test (*U* = 455.50, *p*_adj_ = 0.984, *r_br_* = 0.20). Gender did not differ significantly between groups either (*p*_adj_ > 0.999, *OR* = 1.05, 95% CI [0.36, 3.07]), neither did the prevalence of hypertension (*p*_adj_ > 0.999, *OR* = 1.82, 95% CI [0.53, 6.29]), diabetes mellitus (*p*_adj_ > 0.999, *OR* = 0.74, 95% CI [0.21, 2.61]), smoking (*p*_adj_ = 0.984, *OR* = 2.88, 95% CI [0.81, 10.20]), antiplatelet medication (*p*_adj_ = 0.253, *OR* = 0.27, 95% CI [0.09, 0.85]), or anticoagulant use (*p*_adj_ = 0.410, *OR* = 3.58, 95% CI [1.04, 12.30]), as revealed by Fisher’s exact tests.

To investigate the influence of frailty on the clinical course, group differences in length of stay, the need for ICU, and revision surgery were analyzed. Mann–Whitney U test revealed that the length of hospital stay was longer for the frail (median = 7 days, IQR 4–13) than for the non-frail group (median = 5 days, IQR 4–7), though this difference did not reach significance after correcting for multiple comparisons (*U* = 403.50, *p*_adj_ = 0.410, *r_br_* = 0.30). Similarly, frail patients were more than twice as likely to require postoperative ICU admission and revision surgery compared to non-frail ones (both 41% [n = 9, N_frail_ = 22] vs. 17% [n = 9, N_non-frail_ = 52]), again not reaching the adjusted significance level, as revealed by Fisher’s exact test (both *p*_adj_ = 0.410; *OR* = 3.31, 95% CI [1.09, 10.10]). At discharge, non-frail patients were more likely to return home (76.9% vs. 45.5%, *p*_adj_ = 0.123; inverse relative risk = 2.5, 95% CI [0.2, 0.79]). The overall in-hospital mortality was 2.7%. However, after correction for multiple testing this difference failed to reach statistical significance. See Table 2 for details on clinical outcomes and complications.

**Figure 3:**
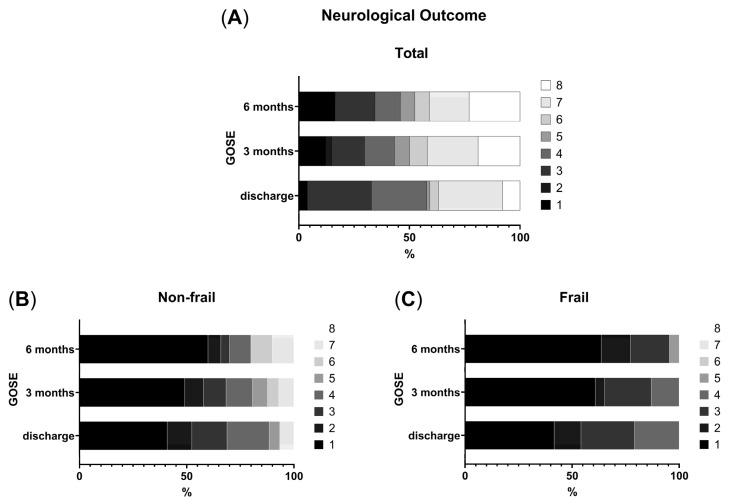
Stacked bar charts display the percentage distribution of GOSE categories at discharge, 3 months, and 6 months. Panel (**A**) shows the total cohort (N = 74 at discharge, N = 72 at 3-month follow-up, N = 64 at 6-month follow-up), panel (**B**) the non-frail subgroup (N = 52 at discharge, N = 50 at 3-month follow-up, N = 44 at 6-month follow-up), and panel (**C**) the frail subgroup (N = 22 at discharge, N = 22 at 3-month follow-up, N = 20 at 6 month follow-up). Each bar represents 100% of patients at the respective follow-up, with gray shades indicating GOSE categories. GOSE = Glasgow Outcome Scale-extended.

The influence of frailty on neurological outcome at discharge, three and six months post-discharge was analyzed applying Mann–Whitney U tests. Results revealed a significantly better outcome for non-frail patients at all time points (frail: median = 3, IQR 3–4; non-frail: median = 7, IQR 4–7; GOSE0: *U* = 170.5, *p*_adj_ = 0.014, *r_rb_* = 0.702; GOSE3: *U* = 61.00, *p*_adj_ = 0.014, *r_rb_* = 0.89; GOSE6: *U* = 55.5, *p*_adj_ = 0.014, *r_rb_* = 0.874). Notably, no frail patient achieved a favorable outcome (GOSE ≥ 6) at any time point (see Figure 3 and Table 3).

Six-month mortality was significantly higher in the frail group (32% vs. 4%, *p*_adj_ = 0.048, relative risk = 3.29, 95% CI [1.67, 5.78]).

See Table 3 and Figure 3 for GOSE scores and long-term outcomes.

### 3.5. Regression Analysis

A binomial logistic regression was performed to determine the effect of frailty, age, antiplatelet, anticoagulation, and revision surgery and predict the likelihood of a good neurological outcome six months post-discharge. The binomial logistic regression model was statistically significant, *χ*^2^ (6) = 61.30, *p* < 0.001, resulting in a medium amount of explained variance, as shown by Nagelkerke’s *R*^2^ = 0.31. Of the five variables entered into the regression model, the following three contributed significantly in predicting outcome: frailty (*p* < 0.001), age (*p* = 0.014), and revision surgery (*p* = 0.009), while the other variables showed no significant effect: antiplatelet (*p* = 0.210) and anticoagulation (*p* = 0.104). Being frail reduced the likelihood of a good neurological outcome, *OR* = 0.02 (95%-CI: [0.00, 0.09]), as did older age, *OR* = 0.92 (95%-CI: [0.87, 0.98]), and revision surgery, *OR* = 0.18 (95%-CI: [0.08, 0.24]).

## 4. Discussion

### 4.1. Frailty Is Associated with Poorer Neurological Outcome

To the best of our knowledge, this study presents the first prospective data evaluating the relationship between frailty and neurological recovery in patients with cSDH. Frailty assessed via CFS significantly correlated with neurological outcome, in terms of higher CFS scores being associated with a poorer neurological outcome. This correlation was stronger than the correlation observed between higher age and poorer neurological outcome. Additionally, we observed a correlation between the CCI and poorer neurological outcome. However, the strength of these correlations was lower than that observed for frailty. These interim findings suggest that while comorbidity burden and higher age contribute to a decreased recovery potential, frailty may provide a more accurate reflection of a patient’s physiological reserve and vulnerability in the context of cSDH.

Frailty represents a distinct, multifactorial syndrome characterized by diminished physiological reserve across multiple organ systems, leading to reduced capacity to cope with stressors and an increased risk of adverse outcomes [16,17,18]. Unlike chronological age, frailty is dynamic and may progress or improve depending on patient-specific factors and interventions [19,20]. Our findings support the hypothesis that frailty is a more meaningful predictor of postoperative outcome than age alone in this patient population.

With rising life expectancy and increasing incidence of falls, the prevalence of frailty is expected to grow, contributing to a further increase in cSDH cases [18,21]. This trend emphasizes the urgent need for better predictors of outcome and patient-centered treatment strategies. Frailty screening could become an useful tool for informing patients and families about anticipated outcomes and shared decision-making [11,22,23,24].

In the subgroup analyses, frail patients with a baseline CFS score equal or higher than five experienced significantly poorer neurological outcomes at both 3- and 6-month follow-ups compared to non-frail individuals. Notably, no frail patient achieved a favorable outcome (GOSE 6–8) at any time point. Frail patients experienced more postoperative complications, including increased ICU admission rates, longer hospital stays, higher revision surgery rates and were less likely to be discharged home. Although these secondary outcome parameters failed to reach statistical significance after correction for multiple testing, our results further underscore the systemic vulnerability of frail individuals and reinforce the need for anticipatory management strategies. These findings complement and extend the existing body of evidence from retrospective studies demonstrating that higher frailty scores correlated with elevated mortality rates in cSDH, higher complication, and recurrence rate [8,11,12,22,23,24,25]. Notably, prospective data are scarce and focused solely on age as risk factor as shown in a larger prospective multicentric trial performed in the United Kingdom in 2017 [2].

In addition to its correlation with postoperative course, the present interim analysis provides important insights into the relative contribution of key predictors of neurological outcome. Notably, frailty demonstrated a strong association, exhibiting a large negative effect compared with revision surgery and chronological age. Our findings suggest that while postoperative factors such as need for reoperation contribute to clinical outcome, baseline physiological vulnerability plays a more decisive role in determining recovery, possible due to an impaired wound healing, reduced vascular stability, and prolonged inflammatory response [26,27,28,29]. Given that recurrence is one of the most impactful complications in cSDH, often requiring repeat surgery and prolonging recovery, frailty may serve as an early predictor of unfavorable postoperative outcome. Tailoring follow-up intensity and surgical technique (e.g., drain duration, use of hemostatic agents) based on frailty status could therefore represent a meaningful advancement in risk-adapted care. Recent evidence suggests that routine repeated CT imaging after treatment of cSDH may not be necessary in all patients [30]. In this context, frailty could serve as a valuable stratification parameter to identify individuals at higher risk of recurrence who might benefit from closer radiological follow-up. Larger retrospective cohorts such as the recently published study by Buwaider and colleagues reported a lower recurrence rate (11%) than our data [31]. They identified male sex, diabetes, midline shift, and antithrombotic therapy and bilateral surgery as independent risk factors for recurrence, while the CCI was an independent risk factor for severe complications. The observed differences in these two studies in recurrence rate may be due to variations in surgical techniques (suction subgaleal drain vs. subdural drain) and the timing of postoperative imaging (4–6 weeks post-surgery vs. 2 weeks post-surgery). Of note, not all patients with recurrence surgery in our cohort were symptomatic. In accordance with our institutional standard operation procedure, progressive mass effect on follow-up CT alone, even in the absence of clinical deterioration, was sufficient to indicate revision surgery. This radiologically guided approach may have contributed to the comparatively high revision rate in our cohort. Furthermore, frail patients often exhibit polypharmacy, including the frequent use of ACE inhibitors, which have been linked to increase risk of hematoma recurrence, representing a potential pharmacological confounder [32,33].

The use of frailty assessment at admission may enhance risk stratification and help guide treatment decisions, particularly in tailoring surgical indications, perioperative care, and follow-up strategies. The better performance of frailty compared to chronological age as a predictor of outcome could support a shift toward more individualized management paradigms in cSDH, potentially informing the decision between invasive surgical intervention and endovascular alternatives. In this context, embolization of the middle cerebral artery (MMA) has emerged as a promising adjunct or alternative treatment option, particularly in patients at increased risk of surgical morbidity. Recent studies suggest that MMA embolization may significantly reduce recurrence rates and the need for repeat surgery [34,35,36]. Frailty could therefore play a pivotal role in identifying patients who may particularly benefit from such less invasive strategies.

Whether the CFS is the most appropriate tool for frailty assessment in neurosurgical populations remains uncertain. While it offers simplicity and practicality, multidimensional approaches incorporating physical, cognitive, and nutritional components may improve predictive accuracy [18,37,38]. However, such tools must remain feasible for routine clinical implementation. Furthermore, since frailty is potentially reversible, interventions such as prehabilitation may play a key role [39,40,41]. The concept of “better in-better out” may be particularly relevant in this context, suggesting that structured preoperative optimization in frail patients could improve postoperative outcomes in cSDH [20,42].

Future research should extend the evaluation of frailty as a predictor of outcome in cSDH to include conservatively managed patients, thereby encompassing the full spectrum of the disease and mitigating potential selection bias introduced by restricting analyses to surgically treated patients. Prospective multicenter studies are warranted to enable larger sample sizes and more robust subgroup analyses, including the evaluation of adjunctive MMA embolization in frail patients. Additionally, long-term outcomes, including functional status and mortality, should be assessed to fully capture the prognostic implications of frailty in cSDH. These approaches could further refine risk stratification and inform individualized management strategies in an aging patient population, particularly given the expected doubling of cSDH incidence by 2030 and the associated rise in surgical and interventional treatments [43].

### 4.2. Limitations

Although the cohort constitutes one of the larger prospective single-center studies in cSDH, the current sample size, particularly within subgroups, is limited and may restrict statistical power. Final conclusions will depend on completion of patient recruitment and follow-up. It should also be noted that our current analysis did not include morphologic imaging parameters such as hematoma architecture or density. As these factors are known to influence recurrence risk, their integration into future analyses of this cohort is warranted to enhance the comprehensiveness of risk profiling [10].

## 5. Conclusions

This prospective analysis highlights the potential role of frailty, measured via the Clinical Frailty Scale, as a strong predictor of postoperative outcomes in cSDH patients. Our results suggest that frailty assessment may offer superior prognostic value compared to chronological age and could serve as a practical tool to support personalized treatment strategies. Validation in larger, multicenter cohorts will be essential to confirm these findings and assess their implications for surgical decision-making and preoperative counseling.

## Figures and Tables

**Table 1:** Demographic characteristics of the study population. Values are presented as median and interquartile range (IQR) or percentage and count, as appropriate. Abbreviations: CFS = Clinical Frailty Scale, CCI = Charlson Comorbidity Index, N = sample size, n = number, and p_adj_ = adjusted significance after Bonferroni–Holm correction for multiple comparison.

Parameter	Total N = 74	Non-Frail (CSF < 5) N = 52	Frail (CSF ≥ 5) N = 22	p_adj_
Gender (%, n)	Male: 69% (n = 51)	Male: 69.2% (n = 36)	Male: 68% (n = 15)	>0.999
Age in years (median, IQR)	76.5 (67.3–82.0)	74.0 (66.0–82.0)	78.5 (72.8–82.8)	0.984
CFS (median, IQR)	3 (2–5)	3 (2–3)	5 (5–6)	
CCI (median, IQR)	4 (3–5)	4 (3–5)	5 (4–7)	
Anti-platelet medication (%, n)	43% (n = 32)	52% (n = 27)	23% (n = 5)	0.253
Anticoagulation medication (%, n)	18% (n = 13)	12% (n =6)	32% (n = 7)	0.410

**Table 2:** Clinical course, postoperative complications, and discharge disposition. Values are expressed as median and interquartile range (IQR) or percentage and count, as appropriate. Frailty was defined as a Clinical Frailty Scale (CFS) score ≥ 5. Abbreviations: ICU = intensive care unit; N = sample size, n = number, and p_adj_ = adjusted significance after Bonferroni–Holm correction for multiple comparison.

Parameter	Total N = 74	Non-Frail (CSF < 5) N = 52	Frail (CSF ≥ 5) N = 22	p_adj_
Length of stay in days (median, IQR)	5 (4–8)	5 (4–7)	7 (4–13)	0.410
Need for ICU (%, n)	24% (n = 18)	17% (n = 9)	41% (n = 9)	0.410
Revision surgery (%, n)	24% (n = 18)	17% (n = 9)	41% (n = 9)	0.410
Discharged home (n, %)	50 (68%)	40 (77%)	10 (46%)	0.123

**Table 3:** Neurological outcomes and mortality after discharge. Neurological recovery was evaluated using (GOSE at discharge, three months, and six months post-discharge (GOSE0, GOSE3, GOSE6). Values are presented as median and interquartile range (IQR) or percentage and count. Abbreviations: GOSE = Glasgow Outcome Scale—extended, N = sample size, n = count, p_adj_ = adjusted significance after Bonferroni–Holm correction for multiple comparison.

Parameter	Total N = 74	Non-Frail (CSF < 5) N = 52	Frail (CSF ≥ 5) N = 22	p_adj_
GOSE0 (median, IRQ)	4 (3–7)	7 (4–7)	3 (3–4)	0.014
GOSE3 (median, IQR)	6 (3–7) (n = 72)	7 (6–8) (n = 50)	3 (1–4) (n = 22)	0.014
GOSE6 (median, IQR)	6 (3–7) (n = 64)	7 (6–8) (n = 44)	3 (1–3) (n = 20)	0.014
Favorable recovery (GOSE 6–8) after six months (n)	34 (46%)	34 (77%)	0	0.002
Death after six months (n)	9 (12%)	2 (4%)	7 (32%)	0.048

## Data Availability

The original contributions presented in this study are included in the article. Further inquiries can be directed to the corresponding author.

## Abbreviations

The following abbreviations are used in this manuscript:

AI	Artificial intelligence
CCI	Charlson Comorbidity Index
CFS	Clinical Frailty Scale
CI	Confidence Interval
cSDH	Chronic Subdural Hematoma
GOSE	Glasgow Outcome Scale—Extended
ICU	Intensive Care Unit
IQR	Interquartile Range
N	Sample Size
n	Number/count
